# Hand Grasping Synergies As Biometrics

**DOI:** 10.3389/fbioe.2017.00026

**Published:** 2017-05-02

**Authors:** Vrajeshri Patel, Poojita Thukral, Martin K. Burns, Ionut Florescu, Rajarathnam Chandramouli, Ramana Vinjamuri

**Affiliations:** ^1^Sensorimotor Control Laboratory, Department of Biomedical Engineering, Chemistry, and Biological Sciences, Stevens Institute of Technology, Hoboken, NJ, USA; ^2^Department of Electrical and Computer Engineering, Carnegie Mellon University, Pittsburgh, PA, USA

**Keywords:** biometrics, human hand, grasping, synergies, principal component analysis

## Abstract

Recently, the need for more secure identity verification systems has driven researchers to explore other sources of biometrics. This includes iris patterns, palm print, hand geometry, facial recognition, and movement patterns (hand motion, gait, and eye movements). Identity verification systems may benefit from the complexity of human movement that integrates multiple levels of control (neural, muscular, and kinematic). Using principal component analysis, we extracted spatiotemporal hand synergies (movement synergies) from an object grasping dataset to explore their use as a potential biometric. These movement synergies are in the form of joint angular velocity profiles of 10 joints. We explored the effect of joint type, digit, number of objects, and grasp type. In its best configuration, movement synergies achieved an equal error rate of 8.19%. While movement synergies can be integrated into an identity verification system with motion capture ability, we also explored a camera-ready version of hand synergies—postural synergies. In this proof of concept system, postural synergies performed well, but only when specific postures were chosen. Based on these results, hand synergies show promise as a potential biometric that can be combined with other hand-based biometrics for improved security.

## Introduction

Identity theft has become a common crime that affects about 7% of the population each year (Harrell, 2015). Passwords and social security numbers are the most common forms of identity verification. Biometrics, or recordable biological measurements, have also been integrated into identity verification systems (Jain et al., 2007). Although there is still much progress to be made in the field (Jain et al., 2006), biometrics eliminates the need for password memorization and offer heightened security. Researched biometrics include palm prints/fingerprints (Jain et al., 2007), iris or retina scans (Hill, 2002), face images (Heo and Savvides, 2012), and electroencephalography signals (Ruiz-Blondet et al., 2016). Recently, researchers have explored the potential of other hand biometrics, including vein patterns (Wang et al., 2008), hand geometry (de-Santos-Sierra et al., 2011), and palm prints (Kumar et al., 2003). The common factor of these identity verification methods is their basis on statically recorded information, usually in the form of a feature matrix, which is then encrypted on a server. While the complexity of feature matrix derivation, encryption methods, and server safety sets the level of security, the fact remains that information can be stolen and used. Recently, Experian, a company commonly used for credit checks and even identity theft protection, was the target of a hack, resulting in the theft of records for approximately 15 million people (Nasr, 2015). This included encrypted social security numbers, passport numbers, and driver’s license numbers. Soon after, a data breach of The United States Office of Personnel Management led to the loss of social security numbers, fingerprints, and other identifiable information, of 21.5 million people (Nakashina, 2015). Moreover, certain biometrics, such as iris scans, can potentially be forged in order to gain entry into biometric-based systems (Ruiz-Albacete et al., 2008). These reports reveal the need for identity verifications systems that do not only rely on static images, scans, or numbers.

Human movement may seem as simple as multiple joints working in parallel to accomplish a task. However, the complete architecture of motor control is still not understood (Scott, 2012). Based on an individual’s anatomy, different neural commands are required to complete the same task across individuals. Furthermore, each individual has advanced his/her motor skills over years of learning. This includes acquisition of basic grasps as an infant to more dexterous motor control such as piano playing and typing. Importantly, these characteristics cannot be forgotten or voluntarily reproduced by another individual. Recently, researchers have taken advantage of the complexity in human movement for use in identity verification systems. Keystroke dynamics involves characterizing keyboard inputs, such as keystroke latencies and durations, finger placement, and finger pressure, to determine a user’s unique typing characteristics (Monrose and Rubin, 2000). Optimized string inputs (i.e., alphanumeric, unstructured vs structured) and classification algorithms have propelled this field of biometrics to commercial use. However, factors, such as emotional state (Epp et al., 2011), keyboard type, and user position, may affect performance (Banerjee and Woodard, 2012). In an attempt to reduce the lengthy time needed to register and identify a user when using keystroke dynamics, Roth et al. (2014) introduced a typing posture biometric that characterizes the shape and position of hands during typing and later introduced keystroke sound (Roth et al., 2015). As a relatively new biometric, these typing characteristics still need to be optimized in order to reduce equal error rate (EER). Arm movement biometrics may be a more appropriate option for individuals without basic typing skills. In-air signatures captured either by camera (Mendels et al., 2014) and smartphones (Casanova et al., 2010; Blanco-Gonzalo et al., 2014) have shown promising results. In an attempt to leverage the complexity of hand movements, in-air signatures of a person’s name (Kamel et al., 2008) or a unique password expressed through American Sign Language (Fong et al., 2013) and touchscreen dynamic (Sae-Bae et al., 2012; Frank et al., 2013) have also been introduced.

Synergy-based movement theory hypothesizes that some commonly used movement patterns are encoded in the central nervous system (CNS). These movement patterns, or synergies, reduce the degrees of freedom that the CNS must control and can be combined to perform more complicated movements. The human hand is one of the most mechanically complex end effectors in the human body and has been researched in relation to synergy-based movement theory for many years. Object grasping is one hand-related activity that is commonly performed throughout the day. It requires coordinated control of four fingers and the thumb to produce postures and force vectors required to grasp and lift objects. It also requires integrating various sensory information (visual, proprioceptive) and planned velocity control (distance and forced dependent) that begins premovement (MacKenzie and Iberall, 1994). It has been found that certain grasping traits maintain high intra-subject similarity and high intersubject variability (Reilmann et al., 2001; Wong and Whishaw, 2004), potentially stemming from different neural and mechanical mechanisms. We and others have previously explored hand synergies and have applied it to motor control models and prosthetics (Santello et al., 2002; Weiss and Flanders, 2004; Vinjamuri et al., 2010; Bicchi et al., 2011). Here, we explore hand synergies’ potential role as biometrics. Often, it is found that the first synergy is characterized by flexion in hand joints, mimicking a power grasp (Santello et al., 2002; Vinjamuri et al., 2010). However, as previously mentioned, motor control is affected by an individual’s unique experience and anatomy. For these reasons, synergy-based biometrics may offer unique advantages compared to static and hand geometry-based biometrics.

In this study, we explore 10 synergies extracted from grasping data for their potential use as biometrics. Each is tested for specificity and sensitivity. We hypothesize that hand synergies contain identifiable information that is robust enough to be incorporated into identity verification systems. As a proof of concept, we also develop a system that can easily be integrated into a camera phone. Subjects pose the end posture of each movement synergy. These 10 “postural synergies” are photographed and tested as potential biometrics.

## Materials and Methods

### Overview

For this study, 10 individuals (5 females, 5 males; 9 right handed, 1 left handed; mean age 21.7 ± 1.95) were recruited under Stevens Institute of Technology Institutional Review Board approval. Subjects performed grasping tasks while wearing a data glove that records hand kinematics. Using principal component analysis (PCA), spatiotemporal synergies were then immediately derived from these data. These spatiotemporal synergies provided us with two forms of synergies that could be used as biometrics: movement synergies and postural synergies. Movement synergies for biometrics were tested using recorded data. Postural synergies were displayed to the subject, who practiced and performed each posture. Postural synergies for biometrics were tested using photographed hand images of these postures. In addition to their own postural synergies, five subjects practiced and performed another subject’s postural synergies to be tested as false entries.

Five of the 10 subjects returned 4–8 months later for a follow-up session of the motion-recording portion of the experiment. This additional dataset was used as additional entry tests for movement synergies.

### Data Capture

Subjects wore a right or left CyberGlove (CyberGlove Systems LLC, San Jose, CA, USA) that records joint angles. For this study, we used 10/18 sensors. These sensors measure the interphalangeal and metacarpophalangeal (MCP) joints of the thumb and MCP and proximal interphalangeal (PIP) joints of the four fingers. Abduction sensors were not used in order to keep replications of a synergy posture simpler. Wrist sensors were not used because they do not pertain to the hand. Data were captured at 125 Hz using a custom-built LabVIEW (National Instruments Corporation, Austin, TX, USA) system. The glove was calibrated for each subject using custom goniometers ranging from −10° to 90°.

**Figure 1:**
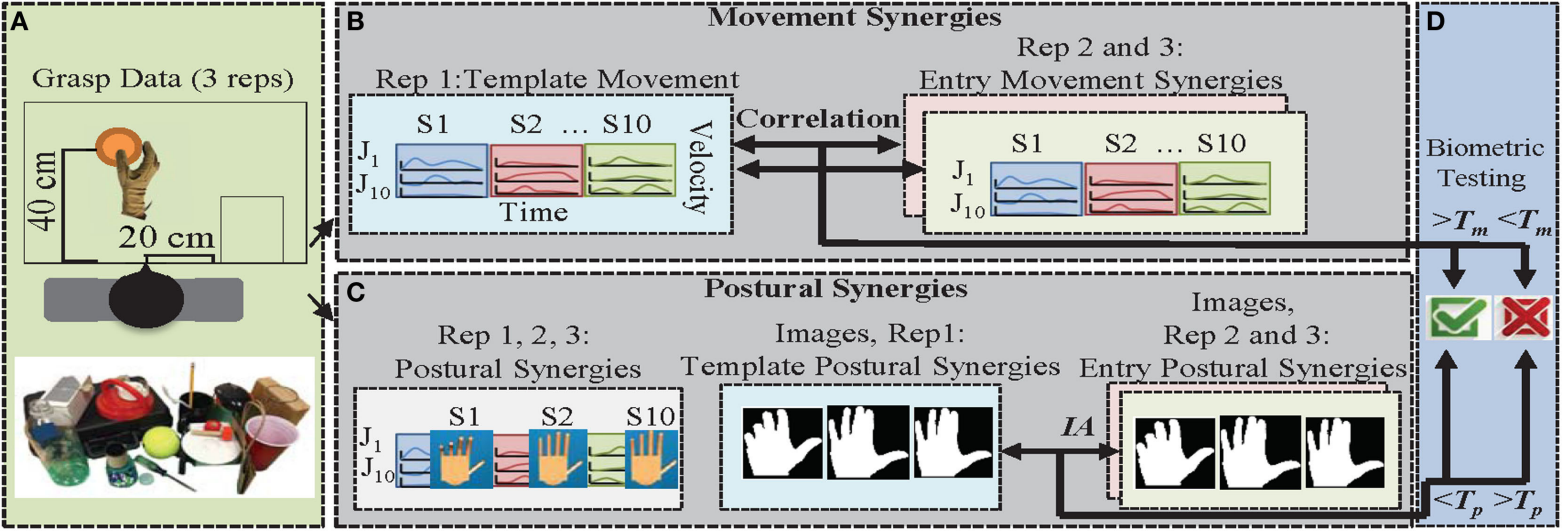
**Procedure overview for movement synergies and postural synergies as biometrics**. **(A)** Twenty-five objects were grasped with three repetitions each. **(B)** Movement synergies (S1, …, S10) were extracted from glove data for 10 joints (J1, …, J10) from repetition 1. Movement synergies extracted from repetitions 2 and 3 were used as entry attempts. **(C)** Immediately after grasp data collection, end position of the movement synergies (derived from repetitions 1–3) were determined and displayed on a virtual hand model. Subjects mimicked these postures and images were taken. Repetition 1 of images was used as a template, and repetitions 2 and 3 of images were used as entry attempts. **(D)** In order to gain access to the system, the correlation between entry and template movement synergies has to be greater than *T*_m_. For postural synergies, the error between entry and template postural synergy images has to be less than *T*_p_.

An overview of the data capture and synergy testing programs is presented in Figure 1. For each subject, the grasping dataset consisted of rapidly grasping 25 objects (3 repetitions) that span 6 types of grasps (power, precision, hook, tripod, lateral key, and spherical). The selected objects were those found in activities of daily living. Each grasp type had four objects associated with it, with the exception of “hook,” which had five objects. The object was placed 40 cm away from the midline of the body, and the hand was placed in an initial resting position 20 cm to the right or left (depending on self-reported hand dominancy) of the body midline (Figure 1A). The subject was asked to rapidly grasp the object after an audio “go” signal and to hold the grasp until an audio “stop” signal was heard. This concluded the grasping portion of the experiment. After data processing, a subject’s postural synergies were shown, after which the subject performed each posture. Images of postural synergies were taken against a green background (for chroma keying) using an 8 megapixel mobile phone camera positioned approximately 38 cm above the hand. During image capture, subjects wore a wrist band to prevent wrist extension/flexion and deviation.

### Synergy Derivation

Synergy-based movement can be generated using a convolutive mixture model (Vinjamuri et al., 2010; Patel et al., 2015). In this model, an impulse originating in higher levels of the CNS passes through a set of linear filters that relate neural and biomechanical structures (possibly cerebellum, basal ganglia, spinal cord, and muscles). Spatiotemporal synergies, represented by joint angular velocity profiles that relate the activity of multiple joints over time, are one form of response from these filters. Here, we consider the rapid grasp condition to inherently contain feedforward direct command signals because they result from minimum or negligible sensory input. Based on previous work, we have found that synergies derived from PCA are able to better capture inherent joint patterns that can reconstruct movements (Patel et al., 2016). Thus, PCA is used to extract the principle components (PCs) of the dataset. Each PC is considered a synergy because it captures a common spatiotemporal pattern that exists across all hand joints.

For each grasp trial, data from movement onset (first time a joint reaches 5% of peak velocity) to grasp completion were extracted (last time 5% of peak velocity is reached). Across all subjects, the maximum time required to complete a grasp was 1.208 s, or 151 samples. Angular velocity profiles were derived from these data to create an angular velocity matrix, *V* [25 × 1,510 (10 joints × 151 samples)]. Singular value decomposition was performed on *V*:

svd(V)=U∑R

such that *U*'*U* is a 25 × 25 identity matrix, *R* is a 1,510 × 1,510 matrix such that *RR*' is a 25 × 25 identity matrix. Σ is 25 × 25 diagonal matrix: diag{λ_1_, λ_2_, …, λ_25_} with λ_1_ ≥ λ_2_ ≥ Λ ≥ λ_25_ ≥ 0. We then reduce matrix *V* to *V˜* by replacing Σ with Σ*_S_*, which contains only the *n* largest singular values, λ_1_, …, λ*_n_*. All the other singular values are replaced by 0s. The approximation matrix *V˜* can be written as

$$\tilde{V}=U_{S}\text{ diag}\{\lambda_{1},\ldots ,\lambda_{n}\}R_{S},$$

where *U_S_* is a 25 × *n* matrix containing the first *n* columns of *U* and *R_S_* is a 25 × 1,510 matrix containing the first *n* columns of *R*. Each column of *R_S_* is called a PC. For the purpose of dimensionality reduction, we perform our analysis on the first 10 principal components, or synergies, only (*n* = 10).

#### Movement Synergies

Spatiotemporal synergies derived using PCA represent movement patterns over time. In order to test movement synergies for biometrics, we derived three sets of synergies, one from each grasping repetition. Each synergy set contains 10 synergies. The first set was used as the “template” that is stored for user registration (Figure 1B). The remaining two sets (entry synergy sets) were used to test the authentication process. The five subjects that were later retested had an additional four entry synergy sets. We used summed correlation across all joints (maximum correlation is equal to 10) to determine the similarity between a template synergy set and entry synergy set. A minimum correlation, *T*_m_, level is required to enter the system (Figure 1D). EER determined by the intersection of false positive rate (false positive/total number of false entries) and false negative rate (false negative/total number of true entries) was used to determine optimum thresholds.

When comparing a template synergy to entry synergy, the following preprocessing steps were performed. Each dataset results in 10 synergies ranked according to the variance they account for. However, this ranking may vary across datasets. Thus, each template synergy was iteratively compared to all synergies from an entry synergy set and then paired with the synergy with highest correlation. After a potential entry synergy is paired with a template synergy, it is removed from the synergy set, so remaining synergies can be paired. Additionally, up to a ±20 sample (160 ms) time shift (zero-padding before or after) was used to account for intersubject time variation. To test correlation by chance, we randomly reshuffled the velocity profile of each joint in a synergy using eight time bins. Each time bin was 20 samples long, with the exception of the last time bin that was 11 samples long. Then, the same comparison approach described above was used to determine correlation by chance.

Each subject’s synergies were also used as false entries for all other subjects. Thus, across all subjects, the performance of each synergy was tested under 40 true conditions (5 subjects × 2 attempts + 5 subjects × 6 attempts) and 180 false conditions (9 false synergies × 2 repetitions × 10 subjects). Note that subjects with four attempts are those who attended the follow-up session. To optimize correlation measurements, we explored the effect of different configurations of the synergy template (i.e., using only certain joints). We tested the effect of joint type (MCP vs PIP) and finger (thumb, index, middle, ring, and pinky). For each “configuration,” correlation values between the adjusted entry synergy and the adjusted template synergy was measured and averaged across subjects. A one-way analysis of variance test, with Tukey–Kramer *post hoc* was used to determine if any configurations significantly increased (*p* < 0.05) correlation. Although this study explores the use of synergies for biometrics, it is important to consider an appropriate time limit for user registration and entry. Thus, we examined the effect of object type and number of objects for potential reduction in data acquisition. When analyzing number of objects, each grasp was first ranked according to the following procedure. First, 25 new synergy sets were extracted, each set omitting a single grasp. For each new set, we determined the average correlation between false synergy sets and the test synergy. Objects that decreased correlation the most were prioritized (ranked highest). After each of the 25 objects were ranked, new synergies were derived by iteratively omitting grasps.

#### Postural Synergies

Postural synergies represent the final position of each movement synergy (velocity profiles are integrated over time to determine final position of each joint). After grasp data were collected, synergies were immediately extracted from all three repetitions (Figure 1C). Each synergy was multiplied by a maximum possible gain under the following criteria: (1) final posture fell within normal range of movement and (2) the majority of a finger did not cross the palm. A virtual hand, built using Simulink 3D Animation toolbox (MathWorks, LLC) was used to display front and side views of the resulting hand posture. Here, we checked whether criterion 2 (from above) was met. Based on preliminary testing, we found that too much flexion in a finger would cause the image analysis procedure to incorrectly omit a finger. Thus, if any of the distal interphalangeal (DIP) joints on the virtual hand cross the upper palm edge, then the weight of the synergy was reduced in 0.01 increments until the DIP joint no longer crossed the palm. Once the synergy postures were finalized by the experimenter, they were shown to the subject. Because subjects were asked to perform these postures for the first time, we allowed an initial practice time for each posture to ensure correct movements (approximately 10 min total). Each synergy was performed and photographed (10 template postures). These were used as template images for each synergy. Then, two more repetitions were taken of each postural synergy (20 entry images). Subjects were encouraged to maintain similarity and “approach” when performing each synergy. “Approach” refers to the order each finger was flexed to achieve the target posture. Additionally, preliminary work showed the thumb to cause excessive variation in images. Thus, subjects were asked to keep the thumb in a natural straight position. In this experimental setting, the hand model in a specific synergy posture was displayed as users attempted to perform each posture. However, in realistic settings, we would expect users to only choose up to three postural synergies to memorize and use for entry.

**Figure 2:**
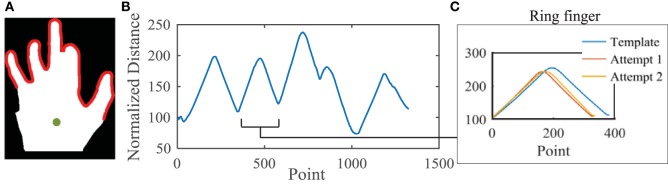
**Image analysis procedure**. **(A)** Preprocessing of the raw image results in a binary figure with the wrist cutoff. The portion being analyzed includes only edges pertaining to digit edges (highlighted in red). The distance between the figure centroid (green) and digit edges is calculated and normalized **(B)**. **(C)** Each finger is separated using peaks and valleys. Here, the ring finger has been separated out. The Euclidian errors between template and entry distances are determined for each finger.

Image analysis of template and entry postural synergies was performed using the Image Analysis Toolbox in MATLAB. In this preliminary work, we controlled lighting to prevent shadows around the hand. Because the focus of this study is to use synergy-based differences across individuals, the effects of other commonly used hand biometrics (skin color, palm/finger size, and vein/texture attributes) were eliminated with the following steps. Preprocessing image analysis steps include: background removal, conversion to binary image (removes skin color and vein/text attributes), wrist cropping, and image centroid calculation. An example of the resulting image is shown in Figure 2A (green dot indicates centroid). Then, the edge of the hand figure that includes only digits portions was taken. Importantly, this outline is a result of MCP and PIP extension/flexion movements, but also reflects natural abduction/adduction movements that occur between fingers. The distance between the palm centroid and each point of the outline is measured and normalized to remove the effect of different hand sizes (Figure 2B). This involves finding the shortest distance from the centroid and dividing all other points from this distance. The resulting values describe a relative hand posture. If an imposter was able to reproduce the exact same ratio of flexion across the fingers as another use, abduction tendencies and enslaving magnitudes, which are unique to an individual, would still cause subtle differences. Each finger is then separated from the outline; this finger profile is used as a basis for comparison (Figure 2C). It should be noted that while the distances are normalized, the number of data points dedicated to a finger is not set. Thus, an individual with a narrower finger or the same individual with a lesser degree of flexion may decrease the number of data points detected for a finger (and *vice versa*). This can be seen 2 C where the blue line represents the outline of the ring finger from the template posture. In entry Attempts 1 and 2, there are fewer data points, possibly resulting from less abduction or less flexion. The Euclidian error between a template finger outline and an entry finger outline is summed across fingers. A maximum error threshold, *T*_p_, is used to determine if the entry postural synergy matches the template postural synergy.

Across all subjects, each of the 10 synergies was tested under 20 true conditions (10 subjects × 2 attempts). Five of the subjects practiced and reproduced postural synergies of another subject; thus, each of the 10 synergies was tested under 10 false conditions (5 subjects × 2 attempts). Because certain fingers of the hand have more dexterity (i.e., index) or enslavement (i.e., ring) than others, we examined which fingers are sources of greater error in true (authentic) and false (imposter) condition. One-way ANOVA was used to compare Euclidian errors between the five fingers. For each individual finger, we then examined whether imposter attempts average equal errors as authentic attempts using a Student’s *t*-test; significance was set at *p* < 0.05.

## Results

### Hand Synergies

**Figure 3:**
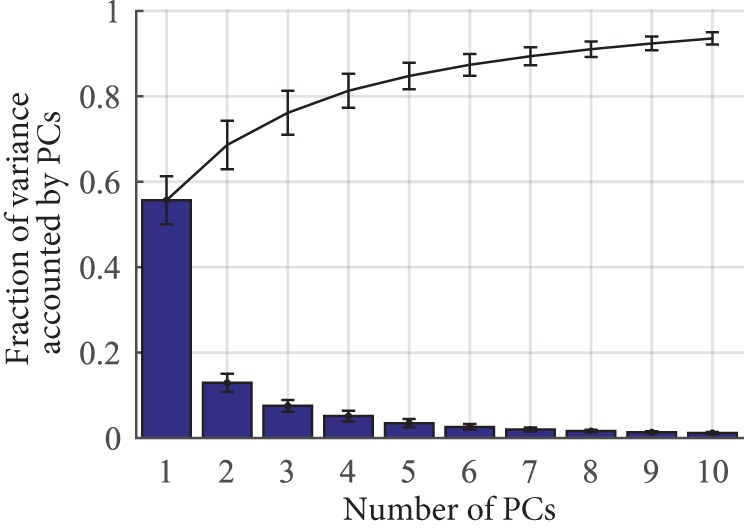
**Bars show the mean fraction of variance accounted by each principle component (PC) across subjects**. Error bars indicate SD. The line plot shows how these variances accumulate from the first PC to the last PC.

**Figure 4:**
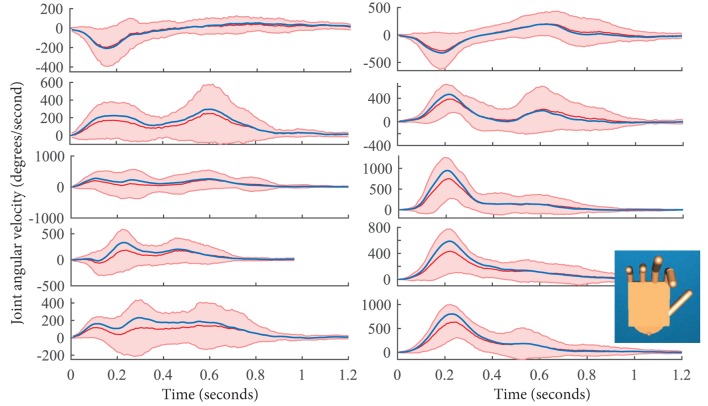
**The red line shows the averaged joint movement across all 25 grasps in one repetition**. SD is provided by the red shaded region. Metacarpophalangeal (MCP) and proximal interphalangeal (PIP) joints of the thumb (T), index (I), middle (M), ring (R), and pinky (P) are shown on the left and right, respectively. To show how the first synergy compares to the average movement, synergy 1 of this particular subject is overlaid by the blue line. Note that this synergy has been multiplied by a gain so that both the red and blue traces match for visual purposes only. The end posture of this synergy is shown on the bottom right. This is considered a postural synergy.

Ten synergies were extracted from grasping data. A movement synergy is defined by velocity profiles for each of the 10 joints. The cumulating fraction of variance accounted for by these synergies is presented in Figure 3. Across all subjects, the first synergy accounted for an average of 54% of the variance. An example of synergy 1 from a representative subject is provided in Figure 4, blue. For comparison, the averaged velocity profile across all 25 grasps is provided with the red trace, with SD depicted by the red shaded regions. Both, the averaged profile and the synergy profile capture the overall pattern. Please note that for visual purpose only, the synergy profile in Figure 4 was multiplied by a gain, such that both the averaged profile and the synergy profile would be on the same scale. As depicted in the figure, there also exists a pattern across and between MCP (left) and PIP (right) joints. The postural synergy representation of this particular movement synergy is shown by the hand image at the bottom right of Figure 4. For movement synergies, each of the 10 velocity profiles needs to adequately match. For postural synergies, images of a hand in these hand configurations need to adequately match.

### Biometric System Based on Movement Synergies

**Figure 5:**
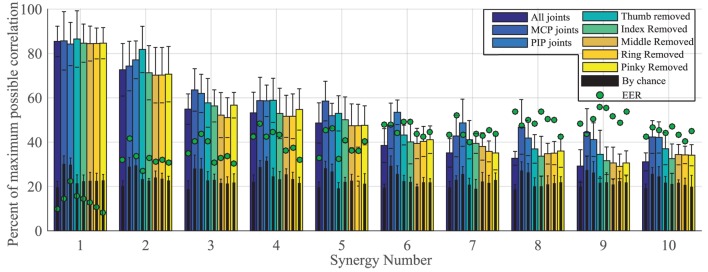
**Across subjects, the mean (bars) and SD (error bars) of correlation values between template and entry movement synergies are shown**. Values are shown for different joint configurations: all joints are used, only metacarpophalangeal (MCP) joints are used, only proximal interphalangeal (PIP) joints are used, thumb joints are removed, index joints are removed, middle joints are removed, ring joints are removed, and pinky joints are removed. Black bars show correlation by chance. Green dots show the calculated equal error rate (EER) for each synergy and configuration. Synergy 1 has the highest correlation and lowest EER values. All synergies had correlations above chance level (black bars).

**Figure 6:**
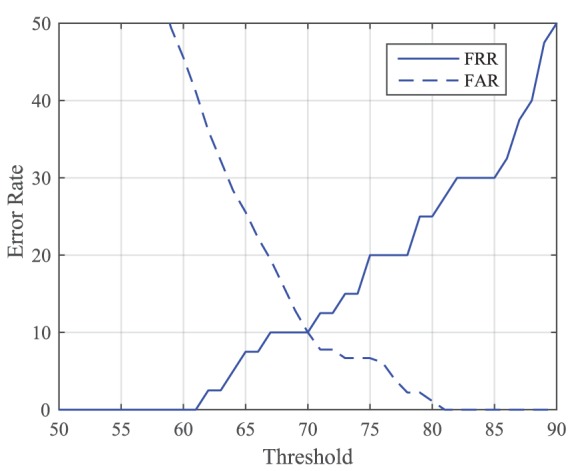
**False rejection rate (FRR) and false acceptance rate (FAR) of synergy 1, when using all 10 joints**. Equal error rate (EER) is calculated at the intersection of these plots. EER is 10% at a threshold of 70% correlation.

To explore the use of movement synergies for biometrics, we first assessed the ability of an individual to reproduce the same synergy profile across repetitions. A maximum of correlation of 100% represents a perfect match. Percent of maximum correlation for different finger configurations (all joints, MCP joints only, PIP joints only, and MCP and PIP joints of each finger removed) across all synergies is shown in Figure 5. All synergies fell above correlation by chance levels (Figure 5, black bars). Results show that the first synergy had the highest correlation, indicating that it was most reproducible across repetitions. Within synergies 1 and 2, no significant difference was found between the different finger configurations; however, removing the thumb resulted in slightly higher correlations. For synergies that did show significant differences across different configurations, *p*-values are provided in Table 1. Namely, higher order synergies (synergies 6–10) had significantly lower correlation scores when more joints were used compared to MCP and PIP configurations that only used five joints each. Green dots in Figure 5 show EER for each synergy and finger configuration. Only synergy 1 fell in an acceptable EER range for biometrics, performing best when pinky MCP and PIP joints were removed (EER = 8.19%) and when all joints were used (EER = 10%). False acceptance rate (FAR) and false rejection rates (FRR) for synergy 1, using all joints is shown in Figure 6. The threshold at the EER point is 70%. Using both synergies 1 and 2 as part of the biometric key did not significantly improve results.

**Table 1:** **Significant differences between different synergy configurations**.

Synergy #	3	6	7	8	9	10
All joints vs MCP	0.3460	0.1483	0.3306	**0.0018**	**0.0012**	**0.0015**
All joints vs PIP	0.6046	**0.0006**	**0.0035**	0.1312	**0.0238**	**0.0019**
MCP vs index	0.5828	0.3727	0.9727	**0.0048**	**0.0114**	**0.0089**
MCP vs middle	0.0816	0.2572	0.8591	**0.0136**	**0.0045**	0.0652
MCP vs ring	**0.0383**	0.4140	0.4518	**0.0171**	**0.0011**	0.0562
MCP vs pinky	0.6412	0.5607	0.3242	**0.0381**	**0.0045**	**0.0492**
PIP vs index	0.8285	**0.0034**	0.1226	0.2390	0.1375	**0.0113**
PIP vs middle	0.2021	**0.0017**	**0.0470**	0.4219	0.0682	0.0788
PIP vs ring	0.1071	**0.0043**	**0.0066**	0.4709	**0.0213**	0.0682
PIP vs pinky	0.8694	**0.0088**	**0.0033**	0.6575	0.0690	0.0599

*MCP, metacarpophalangeal; PIP, proximal interphalangeal*.

*p Values from one-way ANOVA of correlations presented in Figure 5 are given. Only synergies that expressed a significant difference in at least one comparison are provided. Bolded values indicate a p-value < 0.05*.

**Figure 7:**
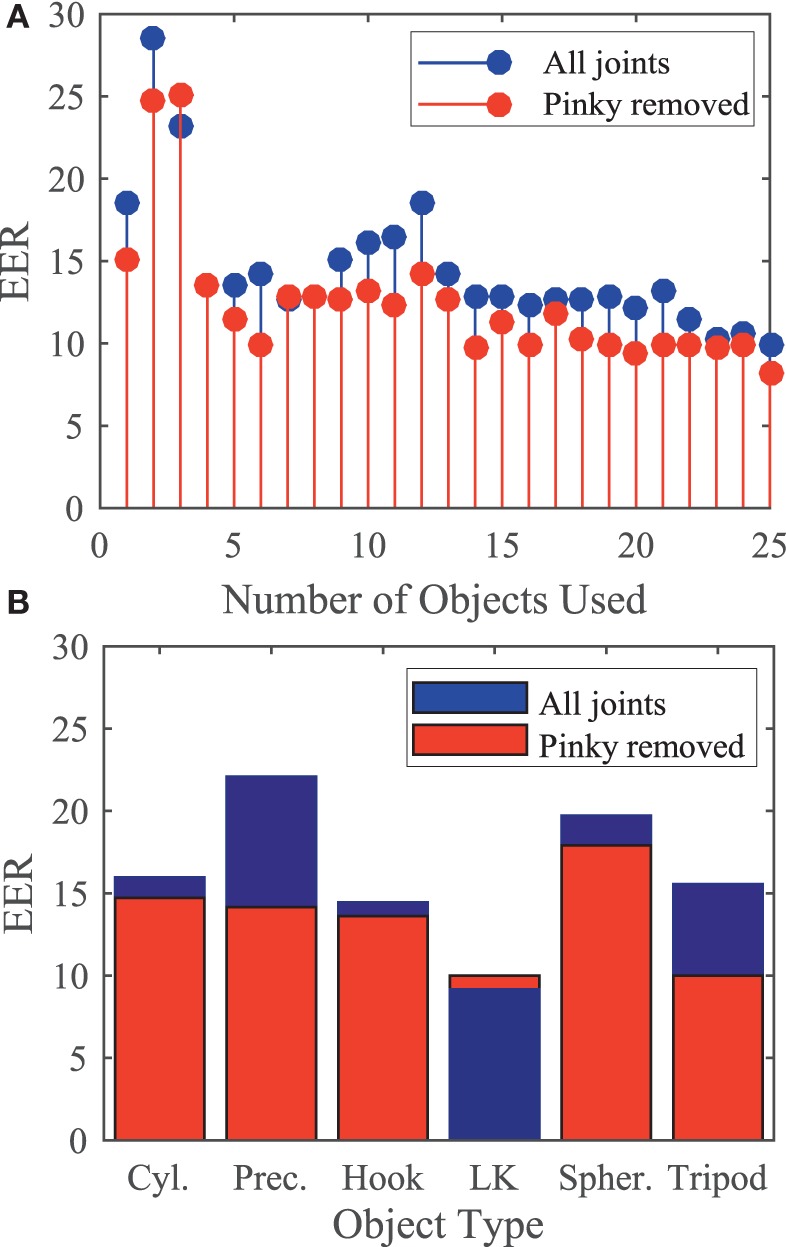
**(A)** Equal error rate (EER) when increasing number of objects are used. Using the first six highest ranked objects produces an EER of 10% when pinky is removed. **(B)** Using only five lateral key grasps produces an EER of 9.17% when the pinky is removed.

In this experimental setup, 25 objects were used to elicit varying grasping patterns. However, in an actual biometrics application, an individual would not be expected to grasp 25 objects. Using EER, we determined the minimum number of objects required to extract and match the first synergy. The overall trend seen in Figure 7A shows increasing the number of objects does not significantly decrease EER. We found that removing the pinky MCP and PIP joints (red) further reduced EER compared to when all joints are used (blue). Synergies extracted from only six of the highest ranking objects produced an EER of 10%. These objects were: screwdriver, water bottle, CD, petri dish, bag handle, and bracelet.

In an attempt to determine if certain grasp types elicit unique hand patterns more robustly, we then extracted synergies from tasks that used certain grasp types. Using these synergies, EER of cylindrical, precision, hook, lateral key, spherical, and tripod grasps is shown in Figure 7B. Synergies extracted from lateral key grasps and tripod grasps showed the lowest EER of 9.17 and 10%, respectively, when the pinky was removed.

### Proof of Concept: Biometric System Based on Postural Synergies

**Figure 8:**
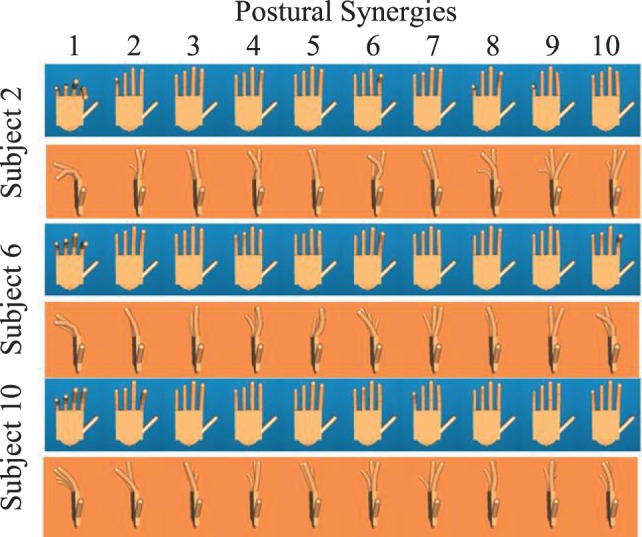
**Front and side views of the 10 postural synergies for subjects 2, 6, and 10 are shown**. Subjects were first trained on performing each of these postures. Then, images of the hand imitating these postures were taken.

For each subject, 10 postural synergies were determined by the end posture of each movement synergy. Figure 8 shows front and side views of these synergies for subjects 2, 6, and 10. Similar to previous research in kinematic hand synergies, the first synergy in all subjects was characterized by flexion in MCP and PIP joints, analogous to a power grasp. Synergy 2 was also characterized by flexion in MCP and PIP joints, but the magnitude at each finger was less consistent across subjects. Synergies 3–9 did not show consistent patterns across fingers and subjects. However, synergy 10 consisted of MCP extension and PIP flexion in all subjects.

**Figure 9:**
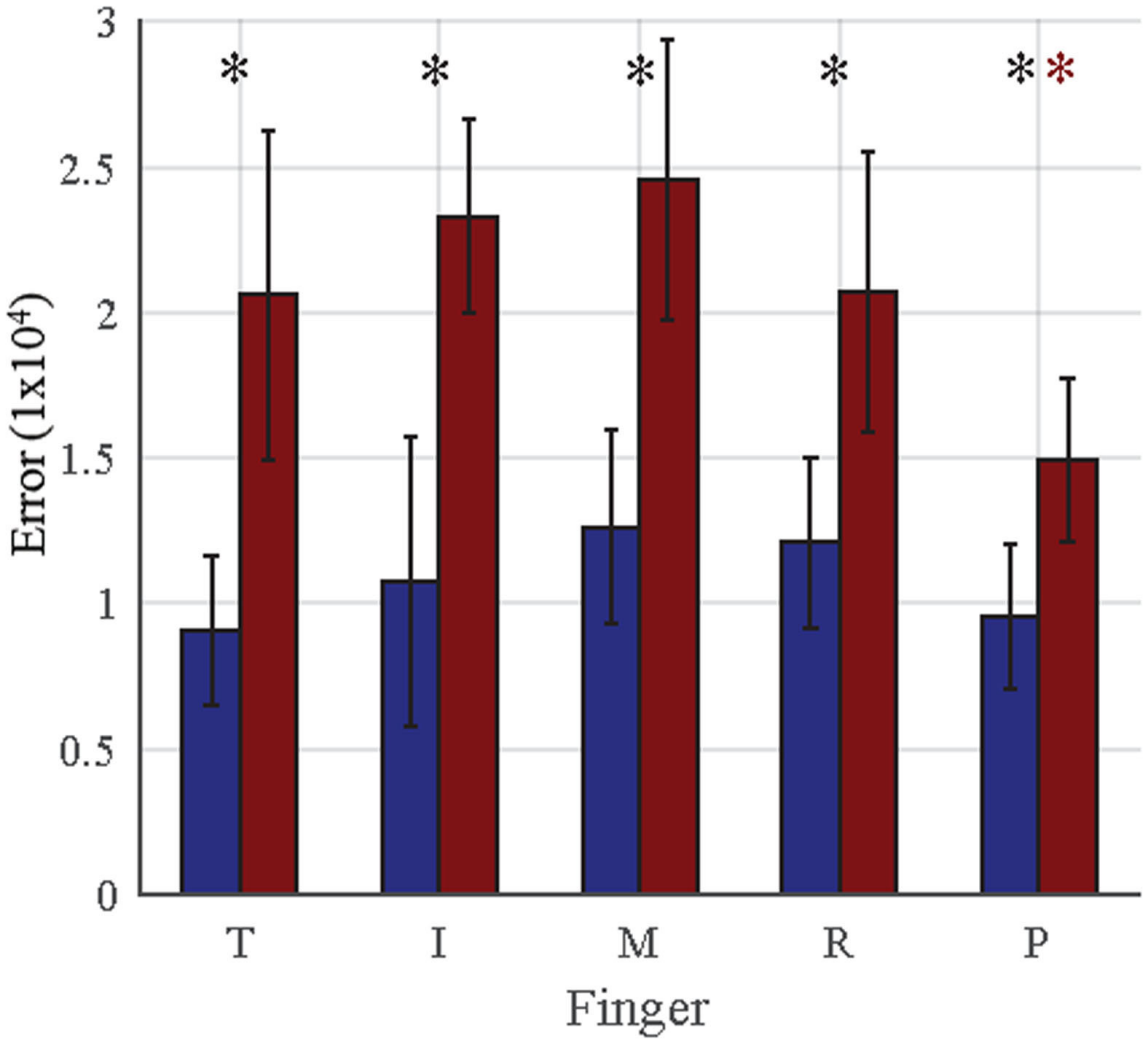
**For each finger, the mean Euclidian error between template and entry postural synergies is shown**. Blue bars represent mean taken across all authentic attempts, and red bars represent mean taken across all imposter attempts. Error bars represent 1 SD. For authentic attempts, no significant differences are seen across fingers. For imposter attempts, the pinky showed significantly less error (indicated by the red star, *p* < 0.05) than all other fingers. In every finger, imposter attempts averaged significantly greater (indicated by the black star, *p* < 0.01) error than authentic attempts.

Although each posture is characterized by MCP and PIP angles for each finger, the imaged posture is evaluated by finger endpoint (i.e., distance of finger peak to palm center). We evaluated the error of each finger across all synergy postures for both authentic (user replicates his/her own posture) and imposter (imposter replicates another user’s posture) cases. Individual finger results are provided in Figure 9. For authentic attempts, no significant differences were found across fingers (one-way ANOVA, *p* > 0.05). For imposter attempts, the pinky (indicated by red star) showed significant differences (one-way ANOVA followed by multiple comparisons using Tukey–Kramer test) compared to the thumb (*p* = 0.0459), index (*p* = 0.0009), middle (*p* = 0.0001), and ring (*p* = 0.0401) fingers. In all fingers (black star), imposter attempts averaged significantly greater errors than authentic attempts: thumb (*p* = 2e−5), index (*p* = 4e−5), middle (*p* = 1.5e−4), ring (*p* = 0.0017), and pinky (*p* = 0.0018).

The performance of using each postural synergy as a biometric key is presented in Table 2. The best performance was achieved by synergy 2 with an EER of 2.5%. We further tested the system by using the first, second, and third best postures (least replication error) from each subject. This resulted in EER of 0, 2.5, 10, and 10% for one, two, three, and four postures, respectively.

**Table 2:** **Equal error rate (EER) for each postural synergy**.

Posture	EER (%)	Posture	EER (%)
Synergy 1	20	Synergy 8	12.5
Synergy 2	2.5	Synergy 9	20
Synergy 3	22.5	Synergy 10	10
Synergy 4	20	Best posture	0
Synergy 5	30	Two best postures	2.5
Synergy 6	20	Three best postures	10
Synergy 7	12.5	Four best postures	10

*EER when each of the 10 postural synergies are used alone as a biometric. Synergy 2 has the best performance with an EER of 2.5%. When the best postural synergy (subject had least error reproducing the posture) was chosen as the biometric key, the system had an EER of 0%*.

## Discussion

### Movement Synergies As Biometrics

All 10 movement synergies were tested as a potential biometric. Results indicate that only synergy 1 is similar enough across repetitions to qualify as a biometric. Synergy 1, accounting for a mean variance of ~54%, characterizes the most general pattern from the grasping dataset. Although it describes a hand-closing pattern for all subjects, results show that each fingers’ flexion rate differs across subjects. Thus, it holds characteristic unique to an individual that can only be determined by grasping everyday objects. Synergy 2 showed some consistency across repetitions but did not perform well enough to be used as a biometric itself. When combining synergies 1 and 2, EER dropped to 9.72%. Further, testing should be done to determine the benefits of including two synergies. Synergies 3–10 were not reproducible enough to be considered for biometrics.

**Figure 10:**
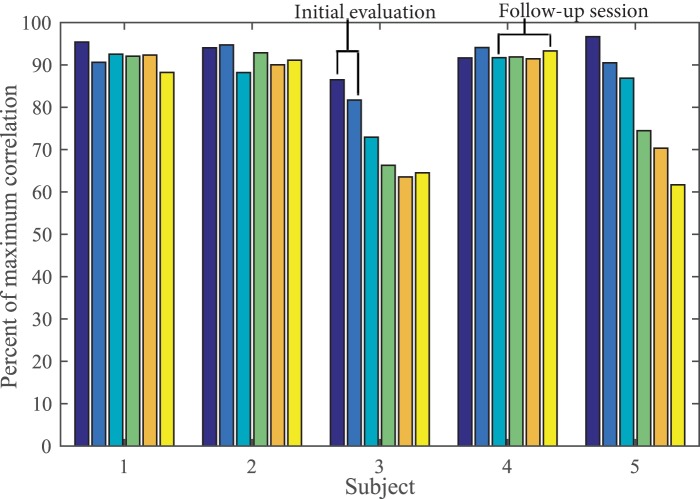
**Short-term stability, from initial evaluation to follow-up session (4–8 months), is seen in subjects 1, 3, and 7, but not subjects 4 and 9**.

Five of the 10 subjects from this dataset were tested 4–8 months after initial evaluation. The present analysis incorporated all attempts when calculating EER, but it is important to consider the effects of short-term stability (or instability) on these values. We used correlation values to measure short-term stability of the five subjects who participated in the follow-up session. Figure 10 shows that subjects 4 and 9 were not able to perform with enough similarity in the follow-up session, while subjects 1, 3, and 7 have consistent performance. Further analysis of these subject’s synergy profiles will help us determine which movement parameters were not reproducible (i.e., peak velocity amplitudes or time domain features).

We expected that eliminating highly individuated digits, such as the thumb or index (Ingram et al., 2008), would decrease EER. However, results showed an increase in EER for synergy 1 (red points in Figure 5) when these digits were not included. Further analysis showed that eliminating the thumb decreased FRR and increased FAR. This indicates that the thumb’s movement is difficult to reproduce within a subject but contributes to the synergy’s uniqueness. Eliminating the index finger slightly increased FRR but did not change FAR. This indicates the index finger is more easily reproducible within a subject. Surprisingly, removing the pinky decreased EER by decreasing FAR. This indicates that imposter attempts were able to reproduce the pinky movement. It has been shown that order of digit contact is often radial to ulnar (Schettino et al., 2013) unless all digits are required for successful grasp (i.e., in cylindrical grasp). In the majority of tasks, the pinky may have followed as a supportive digit, resulting in less complex control.

In terms of choosing optimal objects to decrease system access time, we found that using the six highest ranked objects achieved an EER of 10% (when pinky is removed). As seen in Figure 7, the ranking procedure does not show a clear trend when using one to six objects. The ranking order used in this analysis may not been optimal for a low number of objects because PCA depends on multiple objects. We also found that using the five lateral key grasp objects achieved an EER of 9.17%. Compared to all other grasp types, using lateral key grasps decreased FRR, but increased FAR. This indicates that lateral key grasps were more reproducible within subjects as well as across subjects when compared to the other grasp types.

Our results indicate movement synergies hold unique properties that cannot be reproduced by imposter attempts. In most of the above mentioned scenarios, FAR values were minimal, while FRR values were high; this indicates the sensitivity of the system needs to improve. We believe including additional features that are often maintained in an individual (reaction time, movement time) as well as individualized threshold values will increase performance of the system. Other movement-based biometric systems have used dynamic time warping (DTW) to compare two time profiles. In these studies, testing a pre-determined set of gestures resulted in EER of l.89% (Wu et al., 2013) and 2.58% (Scott, 2012). DTW is currently being evaluated as a potential synergy comparator.

### Postural Synergies As Biometrics

In the postural synergies biometric system, subjects would be required to perform up to three memorized postures in order to gain access to the system. From the 10 postural synergies analyzed, no obvious optimal postural synergy(s) could be determined. EER rates as low as 2.5% show postural synergies have potential as a biometric.

Postural synergies have two layers of uniqueness. First, the movement synergies derived from the grasp data extracts motion patterns that exemplify joint relationship during grasping. Although synergies 3–10 were not reproducible within subjects, they greatly varied across subjects. Second, the ability to conform the hand to the resulting posture, or postural synergy, further exposes patterns in the hand (i.e., unintentional abduction angles resulting from joint flexion). Other posture-based biometric systems employ commonly used postures, such as ASL postures, to spell out a password. In one such study, Fong et al. (2013) concluded that recognizing behavioral patterns is more difficult than recognizing the actual hand shape. If these behavioral patterns are already extracted by the synergies, then further improving the hand shape recognition methods may greatly improve the system. Hand geometry-based studies have used finger width values, variance corrections (de-Santos-Sierra et al., 2011), and hand rotation corrections (Yoruk et al., 2006) to optimize feature extraction and matching performance. Combining traditional geometry-based and intensity-based (hand color) hand biometric methods, we can further improve the postural synergy biometric system.

### Hand Synergies As Biometrics

This study aimed to test the use of movement and postural synergies for use in biometrics. Results are comparable to other movement-based biometric systems that do not use subject-specific passwords (Matsuo et al., 2007; Liu et al., 2009; Bailador et al., 2011) but still below keystroke dynamic performance values (review in Banerjee and Woodard, 2012). Movement synergies reached its best performance at an EER of 8.19%. Postural synergies reached its best performance at an EER of 0 and 2.5%. However, FAR of the system still needs to improve in order to meet adequate performance for biometric use.

Using movement synergies as a biometric requires the use of a hand data glove that would need to be calibrated for all hand sizes. In our procedure, we performed an exhaustive calibration, which would not be ideal for real-world use. We are currently developing a low cost, easy-to-deploy hand data glove that may be more suitable. It is important to keep in mind that this type of biometric cannot be used for rapid user verification. Instead, the user must perform an action in order to gain access in an environment equipped with a computer, data glove, specific grasp objects, etc. Thus, it may only be warranted for higher-security applications that combine multiple biometrics. Like keystroke dynamics and typing posture biometrics, movement synergies do not require the user to memorize a password that has the potential to be forgotten or stolen. However, all three systems require a rather structured setup (i.e., computer to capture data, same keyboard, and similar user positions). Finally, as with all movement related biometrics, template passwords need to be updated due to the effects of aging on motor skills. The postural synergy proof of concept showed that performing postures in front of camera may offer a quicker and more mobile platform. This type of hand biometric can potentially be applied to computers/phones with a camera. Similar to performing in-air signatures, the postures would have to be memorized; however, because of the complexity of synergy hand postures, it may not be necessary to have a private area in order to perform the postures.

Future work includes exhaustively testing both systems with a greater sample size and testing short-term stability and incorporating other commonly used biometric methods such as using averaged time velocity profiles as a synergy, DTW for synergy comparisons, and hand geometry measurements for postural synergies. Finally, we will test whether using the source of hand synergies, i.e., neural signals collected from electroencephalography, can improve performance.

## Ethics Statement

The study was approved by IRB at Stevens Institute of Technology.

## Author Contributions

VP helped design the experiment and recruit subjects, collected and analyzed data, and participated in writing the manuscript. PT helped design the experiment, collected and analyzed data, and participated in writing the manuscript. MB helped create the data collection software and contributed toward revising the manuscript. IF contributed toward statistical analysis. RC contributed toward the design of methods and participated in writing the manuscript. RV directed subject recruitment procedures, developed experiment framework and procedures, and participated in writing and revising the manuscript.

## Conflict of Interest Statement

The authors declare that the research was conducted in the absence of any commercial or financial relationships that could be construed as a potential conflict of interest.
